# Brain-derived uroguanylin as a regulator of postprandial brown adipose tissue activation: a potential therapeutic approach for metabolic disorders

**DOI:** 10.3389/fphar.2025.1569163

**Published:** 2025-04-25

**Authors:** Nikola Habek, Martina Ratko, Dora Sedmak, Ivan Banovac, Vladiana Crljen, Milan Kordić, Marina Radmilović, Siniša Škokić, Martina Tkalčić, Anton Mažuranić, Pero Bubalo, Petar Škavić, Spomenka Ljubić, Dario Rahelić, Aleksandra Dugandžić

**Affiliations:** ^1^ Croatian Institute for Brain Research, School of Medicine, University of Zagreb, Zagreb, Croatia; ^2^ Centre of Excellence for Basic, Clinical and Translational Neuroscience, School of Medicine, University of Zagreb, Zagreb, Croatia; ^3^ Department of Physiology, School of Medicine, University of Zagreb, Zagreb, Croatia; ^4^ Department of Anatomy and Clinical Anatomy, School of Medicine, University of Zagreb, Zagreb, Croatia; ^5^ MKP Ltd., Zagreb, Croatia; ^6^ Institute for Forensic Medicine, School of Medicine, University of Zagreb, Zagreb, Croatia; ^7^ Department of Diabetes, Vuk Vrhovac University Clinic for Diabetes, Endocrinology and Metabolic Diseases, Merkur University Hospital, Zagreb, Croatia

**Keywords:** mouse and human brain, hypothalamus, prefrontal cortex, obesity, type 2 diabetes, glucose homeostasis

## Abstract

**Background:**

Preclinical and clinical research of insulin resistance and glucose homeostasis in metabolic disorders are essential. In this study, we aim to determine the expression of uroguanylin (UGN) in the mouse and human brain, its regulatory mechanisms, and its significance to patients with obesity and type 2 diabetes (T2D).

**Methods:**

UGN expression, regulation, and its correlation with feeding status and obesity in the mouse and human brain were analyzed at the mRNA level using RT-PCR, qPCR, and *in situ* hybridization and at the protein level using Western blot, ELISA, and immunohistochemistry. Brown adipose tissue (BAT) activity was measured using infrared thermography. The volume of interscapular brown adipose tissue in mice was assessed by magnetic resonance imaging.

**Results:**

UGN was expressed in both the mouse and human brain, and its expression was regulated by feeding. In the human prefrontal cortex, UGN was expressed in several interneuron subpopulations across all cortical layers. In Brodmann area (BA) 10, prouroguanylin (proUGN) expression was not regulated by feeding in obesity, whereas this regulation still persisted in BA9. In mice, centrally applied UGN and its analog linaclotide, affecting the hypothalamus, induced both acute and chronic activation of BAT, which decreases the plasma glucose concentration. However, in obesity, proUGN expression was reduced in the human hypothalamus, suggesting reduced postprandial glucose consumption in BAT. Similarly, centrally applied analog of glucagon-like peptide 1 (GLP-1—liraglutide) affected proUGN expression and was associated with increased basal BAT activity but reduced BAT activation after a meal in patients with T2D receiving GLP-1 therapy.

**Conclusion:**

Postprandial BAT activation is regulated by brain-derived UGN, which could serve as a novel therapeutic approach to enhance BAT activity in patients with obesity and T2D to improve postprandial glucose regulation.

## Introduction

Type 2 diabetes (T2D) and its complications represent a significant burden for humanity in the modern world. To reduce the complications of T2D, preclinical and clinical studies on insulin resistance and glucose homeostasis are essential. The function of uroguanylin (UGN) in the brain may play a significant role in the regulation of glucose metabolism and body weight. UGN administered into the brain ventricles of diet-induced obese mice has been shown to decrease the food intake and increase brown adipose tissue (BAT) activity (Folgueira et al., 2016a). BAT, unlike white adipose tissue (WAT), does not store but instead expends energy from fatty acids and glucose. In both humans and rodents, fasting blood glucose concentrations are negatively correlated with BAT activity (Habek et al., 2020; Lee et al., 2010). Magnetic resonance imaging (MRI) studies have shown that patients with prediabetes and T2D exhibit reduced volumes of supraclavicular BAT, further decreasing glucose consumption (Koksharova et al., 2017).

The function of BAT is to increase heat production during cold exposure, which leads to greater glucose consumption and energy expenditure (van Marken Lichtenbelt et al., 2009). Cold-induced BAT activation improves insulin sensitivity in humans (Chondronikola et al., 2014), but this activity is diminished in individuals with obesity and advanced age (Leitner et al., 2017; Ouellet et al., 2011; Pfannenberg et al., 2010; van Marken Lichtenbelt et al., 2009). BAT is also activated after a meal, with glucose uptake in BAT increasing postprandially (Hibi et al., 2016; Vosselman et al., 2013). BAT glucose consumption can occur through insulin-dependent or insulin-independent mechanisms (Orava et al., 2011). In rodents, postprandial activation of BAT is regulated by UGN activity in specific brain regions (Habek et al., 2020). BAT transplantation in mice improves glucose tolerance, enhances insulin sensitivity, reduces bodyweight, and lowers fat mass through daily BAT activation (Stanford et al., 2013). Therefore, a novel strategy for the treatment of obesity and T2D could involve enhancing BAT activity and volume through UGN, thereby improving the postprandial glucose metabolism.

The function and expression of UGN in the brain remain controversial. Plasmatic UGN, released from the intestine after a meal, is primarily present in the form of prouroguanylin (proUGN), whereas the active form, 16-amino acid long UGN, constitutes only 3% of the total amount (Moss et al., 2008). The main question is whether plasmatic proUGN, an 86-amino acid peptide, can pass the blood–brain barrier, be cleaved on the surface of target cells, and activate its receptor, guanylate cyclase C (GC-C), followed by an increase in the intracellular cGMP concentration in the brain cells (Cao and Yang, 2008; Colantuoni et al., 2011; Ratko et al., 2024). GC-C is expressed in various organs, including the intestine, pancreas, regenerating liver, lungs, kidneys, and the male reproductive system. Therefore, circulating UGN could regulate glucose homeostasis by activating peripheral GC-C (Otero et al., 2023; Fernández-Sáez et al., 2023; Folgueira et al., 2016b; Frühbeck et al., 2022). However, UGN-induced regulation of BAT activity relies exclusively on central mechanisms, as GC-C expression has not been detected in mouse BAT (Folgueira et al., 2016a; Habek et al., 2020; Folgueira et al., 2020). GC-C is expressed in many brain regions, including the human prefrontal cortex (PFC), substantia nigra, and cerebellar cortex. In rodents, GC-C is expressed in neurons of the cerebral cortex, amygdala, midbrain, and cerebellum (Dugandzic et al., 2020; Gong et al., 2011; Habek et al., 2021; Mann et al., 2019) and in the hypothalamus, specifically in the arcuate and ventral premammillary nucleus (Merlino et al., 2019). In the arcuate nucleus, GC-C is located in proopiomelanocortin (POMC)-expressing neurons in both rodents and humans. The activation of hypothalamic GC-C and an increase in the intracellular cGMP concentration are proposed to regulate the satiety, BAT activity, and, ultimately, glucose homeostasis (Begg et al., 2014; Dodd et al., 2015; Habek et al., 2020; Valentino et al., 2011).

The presence of the GC-C agonist UGN in the brain has recently been disputed although it is well known that agonists of other guanylate cyclases are expressed in the brain (Kim et al., 2016; Li et al., 2023; McKenzie et al., 2001). Almost 30 years ago, Fan et al. demonstrated UGN mRNA in the opossum cerebellum, midbrain, olfactory bulb, and hypothalamus (Fan et al., 1997). In this study, UGN expression was confirmed at both the mRNA and protein levels in different regions of mouse and the human brain. We found that UGN expression in the brain was regulated by feeding, but this regulation was diminished in individuals with obesity.

## Materials and methods

### Animals

In this study, we used male wild-type (WT) C57Bl/6NCrl and UGN KO mice (littermates, crossbred for generations with C57Bl/6NCrl) aged 2–6 months (ethical approval: 641-01/18-04/01). The UGN KO mice were donated by Dr. Anjaparavanda P. Narren (Cincinnati Children’s Hospital Medical Center, Cincinnati, OH, USA) and were used to determine the specificity of the UGN antibody. For astrocyte isolation, newborn WT mice were used. Mice were fed with standard rodent chow *ad libitum*, with free access to water.

For astrocyte isolation, newborn WT mice (postnatal day 0) were anesthetized on ice and decapitated, and their heads were placed in 70% ethanol. After rinsing the heads in dH_2_O, the skull was opened, the brain was carefully isolated and transferred to Hank’s balanced salt solution (HBSS), the meninges were removed, and the brain was dissected into small pieces. After removal of HBSS, 1 mL of StemPro^®^ Accutase^®^ enzyme solution (Thermo Fisher Scientific, Waltham, MA, USA) was added to the tissue and incubated for 60 min at room temperature with occasional gentle mixing. Enzyme activity was stopped by adding an equal volume of DMEM/F12 medium + GlutaMAX supplement (Dulbecco’s Modified Eagle Medium/Nutrient Mixture F-12 + GlutaMAX™, Thermo Fisher Scientific). The supernatant containing cells was collected and centrifuged for 6 min at 300 g. The supernatant was removed, and the sediment with cells was passed through a magnetically activated cell separation device (Habek et al., 2021; Jungblut et al., 2012). Astrocyte isolation was performed as described by the manufacturer (Anti-GLAST (ACSA-1) MicroBead Kit, Miltenyi Biotec, Inc. Auburn, CA, USA, cat. no. 130-095–826).

### I.n. and i.p. hormone application

Peptides (UGN, insulin, liraglutide, and linaclotide) were administrated intranasally (i.n.) or intraperitoneally (i.p.). After 4 h of fasting, UGN was applied i.n. in a dosage of 10 μg/animal or i.p. in a dosage of 50 μg/animal. Before and at 15 and 30 min, and 1, 2, and 3 h, the activity of BAT was measured. Before and at 30, 60, and 120 min after i.n. application of insulin (10 mU/animal in saline solution, total volume of 5 mL) and GLP-1 analog, liraglutide (30 μg/animal in saline solution, total volume of 5 mL), the interscapular BAT activity was measured. Animals were anesthetized as previously described, and blood and cerebrospinal fluid (CSF) were collected. For purposes of ELISA analysis, the hypothalamus was isolated and quickly frozen in liquid nitrogen. All samples were stored at −20°C until use.

After 4 h of fasting, linaclotide (GC-C agonist) was applied i.n. in dosages of 0.1, 1, and 10 μg/animal. The activity of BAT was measured 1 h after i.n. application, and plasma glucose concentration was measured before and after the experiment.

For the estimation of chronic UGN effects, the activation of BAT after a meal and the volume of interscapular BAT and WAT were estimated in 12 WT and 12 UGN KO male littermate mice. Homozygotes lacking the gene for UGN were crossed with a wild strain of mice (C57Bl/6NCrl strain). The obtained mice were heterozygotes for the UGN gene (F1 generation). Heterozygous animals were crossed for several generations with each other, and their offspring of the appropriate genotype after genotyping were used in further experiments.

Animals were randomly separated into two groups (n = 6). In the control group, i.n. vehicle (saline) was applied two times (5 μL) in the morning (8 a.m.) and afternoon (4 p.m.) for 2 weeks. The same protocol was applied for the experimental group of animals, with a final dosage of UGN (10 μg/animal) dissolved in saline, applied twice daily (5 μL). After 2 weeks of i.n. application, the activation of BAT after a meal and the volume of BAT and WAT were estimated again.

### Human

Human brain tissue was collected from 21 deceased men (mean age: 53 ± 3 years, Supplementary Table S1) with a postmortem delay of less than 24 h. Postmortem analysis revealed no neuropathological abnormalities in brain structures. Obesity was assessed by the forensic physician during regular autopsies. Samples were collected from the arcuate nucleus, substantia nigra, medial cerebellar cortex, and prefrontal cortical Brodmann areas (BA) 9, 10-O, 10-M, 11, and 32 from both hemispheres and stored at −80°C until further use. Less than 200 mL of stomach content was classified as an “empty” stomach. If more than 200 mL of stomach content was found, it was classified as a “full” stomach. Sample collection was approved by deceased’s next of kin by a signed informed consent. Research workers protected personal information and the identity of the deceased, which were not revealed to third persons (Ratko et al., 2024). Experimental procedures were approved by the Ethical Committee of the University of Zagreb, School of Medicine (641-01/18-02/01).

For the purposes of *in situ* hybridization (RNAScope) combined with immunohistochemical analysis, the paraffin sections of the prefrontal cortex were taken from the Zagreb neuroembryological collection. Brain samples were from a 29-year-old male subject with a postmortem delay of 8 h. The subject passed away without experiencing a preagonal phase, and the postmortem interval accurately reflects the onset of neuronal demise. The subject exhibited no prior medical history of neurological or psychiatric conditions, and postmortem analysis revealed no neuropathological abnormalities within the brain structures. Medical history data were sourced from both postmortem reports and medical archives. Information about the subject’s identity and medical histories is securely archived, with brain tissue assigned a code only denoting the subject’s age. The brain tissue underwent segmentation based on Talairach’s coordinates (Banovac et al., 2022; Talairach and Szikla, 1980). Initially, the tissue was fixed in 4% paraformaldehyde (PFA) for 24 h, followed by dehydration in an ethanol gradient (70%, 96%, and 100%), and then incubated in toluene for 24 h. Subsequently, tissue was embedded in paraffin. The prepared tissue was then sectioned into 20-μm-thick coronal slices using a microtome and mounted on VitroGnost Plus Ultra adhesive microscope slides (Biognost, Zagreb, Croatia).

Human participants were patients with type 2 diabetes (T2D), consisting of three women and four men, 61 ± 3 years old, with a BMI of 29.5 ± 0.6 kg/m^2^, mean arterial pressure of 102 ± 3 mmHg, and waist circumference of 100 ± 3 cm. Three were receiving GLP-1 therapy in the form of liraglutide or inhibitors of dipeptidyl peptidase-4 inhibitors, which increase GLP-1 levels. There were no differences in the stated values between the two groups of patients.

### Mouse brain isolation and determination of glucose concentration

To determine changes in UGN expression after a meal, animals were let to fast overnight and were fed with standard rodent chow on the day of the experiment. Animals were sacrificed by cervical dislocation. To quantify mRNA expression, brain samples and plasma were collected before (after overnight fasting) and 2, 4, and 6 h after a meal. For determining UGN expression at the protein level, tissue was collected in overnight fasting conditions, 30 min, and 2 h after a meal. All efforts were made to reduce the number of animals in this study and minimize animal suffering.

Before brain isolation, blood glucose concentration was determined from the tail blood sample. The animal was anesthetized by i.p. application of ketamine/xylazine (80–100 mg/kg and 10–12.5 mg/kg, respectively). Glucose concentration was measured in CSF samples obtained by a puncture of the *cisterna magna*. The CSF samples with visible blood traces were discarded. The animal was placed in a stereotaxic apparatus, and a skin incision was made on the back of the neck. By spreading the muscle and connective tissue, the *cisterna magna* was accessed, and a puncture was performed with a glass micropipette. Plasma and CSF glucose concentrations were determined using a ContourXT glucometer (Ascensia Diabetes Care Holdings AG, Basel, Switzerland).

When sampling the brain for *in situ* hybridization and immunohistochemistry analysis, the animal was first transcardially perfused with 0.01 M PBS (phosphate-buffered saline), followed by 4% PFA in PBS, for fixation. The brain was removed and incubated for 16 h in 4% PFA for post-fixation. The next day, the brain was first washed in PBS and then cryoprotected in a sucrose gradient (10%, 20%, and 30%, 24 h each step), embedded in Tissue-Tek O.C.T. Compound (Sakura Finetek USA, Inc., Torrance, CA, USA), quickly frozen, and stored at −20°C. Brain sections were cut on a cryocut and mounted on SuperFrost Plus adhesion slides (Fisher Scientific, Loughborough, United Kingdom).

For RNA and protein isolation, mice were transcardially perfused with PBS to avoid possible false-positive UGN expression in the brain due to detecting blood UGN. The brain and proximal part of the small intestine were isolated, and the region of interest were dissected and frozen in liquid nitrogen. Tissue samples were stored at −80°C before use. For Western blot analysis, animals fasted overnight before tissue samples were collected.

### Reverse transcription polymerase chain reaction (RT-PCR)

Mouse brain tissue (cortex, cerebellum, midbrain, and hypothalamus) and the proximal part of the mouse small intestine were weighed. TRIzol reagent (Thermo Fisher Scientific) was added (1 mL per 100 mg of tissue or per 10^5^–10^7^ isolated astrocytes). After homogenization on the ultrasonicator (Microson Ultrasonic Cell Disruptor, Misonix, Farmingdale, NY, USA), the homogenates were incubated for 5 min at room temperature, and 0.2 mL of chloroform per 1 mL of TRIzol reagent was added, incubated for 2 min at room temperature, and centrifuged for 15 min at 12,000 g at +4°C. The upper, aqueous phase with RNA was carefully transferred into new test tubes, and 0.5 mL of isopropanol was added per 1 mL of used TRIzol reagent and incubated for 10 min at room temperature. The mixture was then centrifuged for 10 min at 12,000 g at +4°C. The RNA precipitate was washed with 1 mL of 75% ethanol, and the samples were again centrifuged for 5 min at 7,500 g at +4°C. The RNA precipitate was briefly dried; then, it was placed in a vacuum concentrator (Concentrator plus, Eppendorf, Hamburg, Germany) for 5 min to remove excess ethanol. We dissolved the total isolated RNA in dH_2_O and either immediately used it for transcribing into cDNA or stored it at −80°C until further use.

RNA concentration was measured on a NanoDrop ND-1000 spectrophotometer (Thermo Fisher Scientific). An amount of 1 μg of total RNA was transcribed into complementary DNA (cDNA) using the GoScript™ Reverse Transcription System (Promega Corporation, Madison, WI, USA) according to the following protocol: 1 µg of RNA, 1 µL of Oligo(dt) primer (500 μg/mL), and H_2_O in a total volume of 5 µL was heated at 70°C for 5 min and quickly cooled on ice for 5 min. An amount of 4 μL GoScript (5x) reaction buffer, 2 µL MgCl_2_ (25 mM), 1 µL nucleotide mixture (10 mM), 0.5 µL ribonuclease inhibitor (40 U/µL), 1 µL GoScript reverse transcriptase, and 6.5 µL H_2_O were added to the reaction. The PCR protocol was as follows: 5 min at 25°C, 60 min at 42°C, and 15 min at 70°C. We stored the synthesized cDNA at −20°C until use.

The primers used in this analysis of GN and UGN expression were as follows: UGN: S: 5′GGT​GGC​AGG​CAG​GTG​GAC​A 3'; AS: 5′CTG​GGA​GGA​TGG​CGA​TTA​CTT​CA 3'; GN: S 5′TTGGCT GTCCTGGTAGAAG 3′, AS 5′TGT​GGC​AGG​GCA​ATA​GAT​G 3'; and GAPDH: S 5′ ACG​GCC​GCA​TCT​TCT​TGT​G 3′, AS 5′ CCC​ATT​CTC​GGC​CTT​GAC​TG 3’.

The PCR mixture consisted of the following: 1 µL of each desired primer (10 pM), 0.5 µL nucleotide mixture (10 mM), 5 µL reaction buffer (5x), 1 µL MgCl_2_ (25 mM), 0.125 µL GoTaq polymerase (5 U/µL), and 13.375 µL dH_2_O. The PCR protocol was as follows: 2 min at 94°C, 30 s at 59.8°C for UGN and 55.8°C for GN, and 1 min at 72°C (1 cycle); 30 s at 94°C, 30 s at 58.8°C or 55.8°C, and 1 min at 72°C (30 cycles). The products of the PCR were analyzed electrophoretically on a 1.8% agarose gel containing the nucleic acid dye GelRed^®^ (Biotinum, Fremont, CA, USA) and sequenced to confirm specificity.

### Quantitative real-time PCR (qPCR)

UGN expression at the mRNA level was determined in the cortex, hypothalamus, and cerebellum before a meal and at 2, 4, and 6 h after a meal. All required reagents were obtained from the TaqMan Real-Time PCR Assay (Thermo Fisher Scientific). We used assays for *Guca2b* (proUGN: Mm01192051_m1, UGN) and Actb (β-Actin) (Mm00607939_s1, ACTb) as a housekeeping gene. The results were obtained using 7500 Real-Time PCR System (Thermo Fisher Scientific). Expression normalized to Actb is calculated by powering number 2 to the difference between the threshold cycle of the gene of interest and Actb (2^−ΔCt^) and presented changes in expression 2, 4, and 6 h after a meal compared to that during fasting (0 h) conditions. The product of the PCR was confirmed by gel electrophoresis.

### *In situ* hybridization (RNAScope)

Human paraffin prefrontal cortex sections (20 µm) were first submerged in PBS and photobleached for 48 h under strong LED light at +4°C in order to reduce autofluorescence. Sections were then deparaffinized, washed in PBS, incubated in 3% H_2_O_2_ for 15 min to inactivate peroxidase activity, and then washed three times for 10 min in PBS. Mouse sagittal brain sections (10 µm) were washed three times for 10 min in PBS, incubated in 3% H_2_O_2_ for 15 min to inactivate peroxidase activity, and washed three times for 10 min in PBS.

*In situ* hybridization combined with immunofluorescence analysis was performed using the RNAscope Multiplex Fluorescent V2 Assay Kit (cat. no. 323110, Advanced Cell Diagnostics a Bio-Techne Brand, Newark, CA, USA). All chemicals and reagents were obtained from the kit, unless otherwise stated. Human and mouse brain sections were first pretreated with target retrieval buffer (cat. no. 322001) for 10 min at 95°C and then cooled down to room temperature and washed five times for 10 min in self-prepared PBS-DEPC (diethyl pyrocarbonate, Sigma-Aldrich, St. Louis, MO, USA) buffer. The barrier was drawn by a ImmEdge™ hydrophobic barrier pen (Vector Laboratories, Inc., Newark, CA, USA) and left to dry for 40 min at room temperature. Sections were than pretreated with Protease III (cat. no. 322331) for 30 min at 40°C and washed five times for 10 min in PBS-DEPC. Afterward, human sections were hybridized with RNAScope probes for UGN mRNA (Hs-GUCA2b, cat. no.: 433011) and GAD1 mRNA (Hs-GAD1-C2 cat. no.: 404031-C2), and mouse sections were hybridized with a probe for UGN mRNA (Mm-*Guca2b*, cat. no. 428001). The hybridization step was performed for 2 h at 40°C. Sections were then washed for 2 min in 1x washing buffer (cat. no. 310091). The next step was amplification, which consisted of incubation with amplifiers in the following order: AMP1 for 30 min at 40°C, wash for 2 min 1x wash buffer at room temperature, AMP2 for 15 min at 40°C, wash for 2 min 1x wash buffer at room temperature, AMP3 for 30 min at 40°C, and wash for 2 min 1x wash buffer at room temperature. Following this was the staining step: C1 channel staining (UGN mRNA probe) sections were incubated in HRP (horseradish peroxidase)-C1 activator for 15 min at 40°C, washed for 2 min in 1x wash buffer, stained with fluorescein or Cy3 (1:100) Tyramide Signal Amplification (TSA™) (Perkin Elmer, Waltham, MA, USA.) for 30 min at 40°C, washed for 2 min in 1x wash buffer, and incubated in HRP blocker for 15 min at 40°C; C2 channel staining (GAD1 mRNA probe) sections were incubated in HRP-C2 activator for 15 min at 40°C, washed for 2 min in 1x wash buffer, stained with Cy5 TSA™ (1:100) (Perkin Elmer) for 30 min at 40°C, washed for 2 min in 1x wash buffer, and incubated in the HRP blocker for 15 min at 40°C.

Simultaneous immunohistochemical analysis of interneuron subpopulations was performed on human brain sections. Sections were washed three times for 10 min in PBS and then incubated in a blocking solution containing 0.01 M PBS, 0.3% Triton (Sigma-Aldrich), and 3% donkey serum for 1 h at room temperature. Primary antibodies used were mouse monoclonal anti-Calretinin 1:1,000 (cat. no. CR6B3, Swant AG, Burgdorf, Switzerland), mouse monoclonal anti-Calbindin 1:10,000 (cat. no. D-28, Swant), and rabbit polyclonal anti-Parvalbumin 1:1,000 (cat. no. Ab11427, Abcam, Cambridge, United Kingdom). Primary antibodies were incubated in the blocking solution overnight at 4°C. The next day, sections were washed three times for 10 min in PBS. Secondary antibodies used were donkey anti-mouse Alexa Fluor 488 1:500 (cat. no. 150105, Abcam), donkey anti-mouse AlexaFluor 555 1:500 (cat. no. 150110, Abcam), and donkey anti-rabbit AlexaFluor 555 1:500 (cat. no. 150074, Abcam). Incubation with secondary antibodies was performed in PBS for 2 h at room temperature, followed by washing three times for 10 min in PBS.

At the end of each protocol, human and mouse sections were stained with DAPI (4′,6-diamidino-2-phenylindole) 1:4,000 (Thermo Fisher Scientific) for 5 min at room temperature, washed three times for 10 min, and mounted using the VECTASHIELD^®^ Antifade Mounting Medium (Vector Laboratories).

The fluorescent signal acquisition was performed on the FV3000 confocal laser scanning microscope (Olympus Corp., Shinjuku, Tokyo, Japan). Fluorescein TSA dye and Alexa Fluor 488 antibody signal were excited by a 488-nm laser line, and fluorescent signals were collected at 500–540 nm. Cy3 TSA and Alexa Fluor 555 antibody signals were excited by a 546-nm laser line, and fluorescent signals were collected at 560–576 nm. Cy5 TSA signal was excited by a 640-nm laser line, and fluorescent signals were collected at 650–750 nm. DAPI signal was excited by a 405 nm laser line, and fluorescent signals were collected at 430–470 nm.

### ELISA

UGN expression at the protein level (proUGN) was determined with the Enzyme-Linked Immunosorbent Assay kit (for human brain samples: E2738Hu, Bioassay Technology Laboratory, Birmingham, United Kingdom; for animal samples: MBS912299, MyBioSource, San Diego, CA, USA) following the manufacturer’s instructions.

Frozen human tissue samples were thawed on ice and washed in ice-cold PBS. The following desired areas of the prefrontal cortex (PFC) were sampled: BA9 from the dorsal part of the superior frontal gyrus, BA10-O from the ventral (orbital) aspect of the frontal pole, BA10-M from the ventromedial part of the superior frontal gyrus, BA11 from the rostral part of the straight gyrus, and BA32 from the paralimbic cortex situated between the paracingulate sulcus and the cingulate gyrus.

Isolated mouse and human brain tissue were homogenized in ice-cold PBS (1g tissue = 9 mL PBS) with an ultrasonic processor (Q55 Sonicator^®^, QSonica Sonicators, Newton, CT, USA). The homogenate was centrifuged (5 min, 5,000 g, +4°C; Eppendorf Centrifuge 5415 R, Hamburg, Germany). The supernatant was stored at −80°C until use.

Human brain samples (40 µL) were incubated with anti-UGN antibody and streptavidin-HRP for 60 min at 37°C (Heratherm™ Compact Microbiological Incubator, Thermo Fisher Scientific). After 5x 1-min washes, substrate solutions A and B were added, followed by incubation for 10 min at 37°C. Optical density was read using a microplate reader (GloMax^®^ Explorer Multimode Microplate Reader, Promega Corporation) set at 450 nm.

Animal samples were pipetted into wells pre-coated with a proUGN-specific antibody and incubated for 2 h at 37°C (Heratherm™ Compact Microbiological Incubator, Thermo Fisher Scientific, Promega Corporation, Madison, WI, USA). After removing excess liquid, samples were incubated with a biotin antibody for 1 h at 37°C and then washed three times. HRP-avidin was added to the wells for 1 h, after which the washing steps were repeated. A 30-min incubation period with the substrate in the dark was interrupted with a Stop solution, and the absorbances were read with a microplate reader (GloMax^®^ Explorer Multimode Microplate Reader) at 450 nm. The control group was analyzed on the same ELISA plate as the experimental groups to avoid potential inter-assay variation. The intra-assay coefficient of variation for human samples was 9.42, which is below the recommended threshold of 10.

### Western blot

Mouse brain tissue was thawed at +4°C. Tissue was weighed and homogenized on ice with an ultrasonicator in a cell lysis buffer consisting of 20 mM Tris, 150 mM NaCl, 1 mM EDTA, 0.5 mM Na_3_VO_4_, 50 mM NaF, 1 mM Na_2_MoO_4_ x 2H_2_O, 5 mM Na_4_P_2_O_7_ x 10 H_2_O, 1% Triton X-100, 0.5% Na-deoxycholate, 0.1% sodium dodecyl sulfate (SDS), and 10% glycerol. The homogenate was centrifuged for 10 min at 12,000 g, and the supernatant was transferred to a new microtube. Protein concentration in each sample was measured by the Bradford photometric method on a Visible Spectrophotometer M201 (Spectronic CamSpec Ltd., Leeds, United Kingdom) at 595 nm.

Protein samples for electrophoresis were prepared as follows: the sample contained 100 µg of protein, 5 µL of sample buffer with 2 µL of reducing agent (dithiothreitol, DTT) (Thermo Fisher Scientific), and H_2_O to a volume of 20 µL. The samples were then boiled at 95°C for 5 min. The acrylamide/bisacrylamide running gel (16%) and stacking gel (4%) were used. The running buffer consisted of 0.025 M Tris base, 0.15 M glycine, and 0.1% SDS. The prepared samples (20 µL) were placed in individual wells. Electrophoresis was performed at 120 V for 2 h.

Proteins were transferred to a PVDF membrane with pores of 0.2 µm to better adsorb proteins of low molecular mass as the molecular mass of proUGN is 12 kDa. The transfer buffer contained 0.02 M Tris, 0.14 M glycine, and 20% methanol. Protein transfer was performed at 10 V overnight at +4°C. The membrane was washed for 5 min in PBS +0.1% Tween-20 (PBSt) and incubated in a blocking buffer containing 5% nonfat milk powder in PBSt for 1 h at room temperature, followed by an incubation with primary antibodies (1:500, cat. no. 18113-1-AP, Rabbit Anti-proUGN, Proteintech Group, Manchester, United Kingdom and 1:2,500, cat. no. sc-32233, Monoclonal mouse Anti-GAPDH, Santa Cruz Biotechnology, Inc., Heidelberg, Germany) overnight at +4°C. The membrane was washed three times for 10 min in PBSt. The membrane was incubated with secondary antibodies labeled with HRP in blocking buffer (1: 10,000 Anti-rabbit HRP, A-27011, Thermo Fisher Scientific and 1:7,500 cat. no. 715-035-150 Jackson Immunoresearch, Cambridgeshire, United Kingdom) at room temperature for 1 h. The membrane was then washed three times for 10 min in PBSt and incubated in the SuperSignal West Femto substrate (Thermo Fisher Scientific). The signal was visualized on a ChemiDoc XRS + device (Bio-Rad Laboratories, Hercules, CA, USA). The image before the first saturation of the signal was taken for analysis. Analysis was performed in the Image Lab Software (Bio-Rad Laboratories).

### Immunohistochemical analysis

Wild-type and UGN KO mice were anesthetized as previously described and transcardially perfused with PBS and 4% PFA. Brains were isolated as previously described and incubated in 4% PFA overnight. Brain tissue was cryoprotected with an ascending series of sucrose solutions (10%, 20%, and 30% in PBS, each overnight at +4°C). Sections of the regions of interest were cut frontally or sagittally at a thickness of 4 µm (−25°C, Cryostat 2300 slicer, Leica Biosystems, Wetzlar, Germany) and deposited on positively charged glass slides (Superfrost plus, Thermo Fisher Scientific). The slides were stored at +4°C until use.

Tissue was rehydrated for 10 min in 0.01 M PBS. The antigen retrieval procedure was performed by boiling the slides in 0.01 M citrate buffer (pH = 6) for 20 min. The samples were then allowed to cool to room temperature and permeabilized for 15 min in 0.2% Tween 20 in 0.01 M PBS. After permeabilization, the samples were washed twice for 5 min in PBS and placed in a blocking buffer consisting of 1% bovine serum albumin (BSA) in PBS. Antigen blocking lasted for 1 h at room temperature. The samples were then incubated in specific primary antibodies (1:50, cat. no. 18113-1-AP, Rabbit anti-proUGN Proteintech 1:25, cat. no. sc-34428, Goat Anti-GC-C Santa Cruz) at +4°C overnight. After incubation in the primary antibody, the samples were washed three times for 10 min in PBS. Samples were incubated with secondary antibodies (1:500 cat. no. ab150073, Anti-rabbit Alexa Fluor 488 or ab150075, Anti-rabbit Alexa Fluor 647 Abcam and 1:500 cat. no. A-11055 Anti-goat Alexa Fluor 488, Thermo Fisher Scientific) in a blocking buffer (1 h at room temperature). Fluorescently stained samples were incubated in DAPI (1:4000, Thermo Fisher Scientific) for 5 min and washed for three times for 10 min each and covered with a glass coverslip in a medium for preserving fluorescence. The fluorescent signal acquisition was performed on an Olympus BX51 fluorescent microscope (Olympus Corp).

### MR imaging and data acquisition

Magnetic resonance imaging was performed on a Bruker BioSpec 70/20 USR system with Paravision 7.0 software (Bruker BioSpin, Germany) in a Tx/Rx configuration using an 86-mm volume coil (MT0381, Bruker BioSpin, Germany) for transmitting (Tx) and a 2-element mouse brain surface coil (MT0042, Bruker BioSpin, Germany) for receiving (Rx) (Branca et al., 2013; Cassidy et al., 2009; Habek et al., 2020; Rasmussen et al., 2013).

Mice were anesthetized in an induction chamber filled with a mixture of 70/30% N_2_/O_2_ containing 4% isoflurane (Abbott, United Kingdom) and maintained in the anesthetized state during scans by continuous delivery of 1%–1.5% isoflurane in a 70/30% N_2_/O_2_ mixture. Anesthesia was controlled by maintaining the respiratory rate at 80–100 breath/min, measured *via* an optical sensor. Body temperature was recorded using a MR-compatible rectal temperature probe and maintained at 37°C ± 0.2°C by a feedback-controlled warm water circulation system (Medres, Cologne, Germany).

The animals were placed in the Bruker MRI mouse cradle in a supine position, with the receive coil placed over the interscapular region. Neither cardiac nor respiratory gating was used during acquisition.

First, axial and sagittal reference anatomical images were acquired with a turbo spin-echo sequence (TurboRARE), with repetition time (TR)/echo time (TE) of 1,000 m/10 m and a RARE factor of 4. A total of 15 slices were acquired in each scan at an in-plane isotropic resolution of 140 µm (matrix 250 px x 100 px, field of view 35 mm × 14 mm), with slice thickness and inter-slice gap of 1 mm and 0.5 mm, respectively. The reference scan duration was 25 s.

For the fat fraction measurements, two identical high-resolution turbo spin-echo proton density weighted (PDw) sequences were used with the following common scan parameters: TR = 5,000 m, TE = 7.5 m (echo spacing 3.75 m and RARE = 4), 21 slices, 0.7 mm slice thickness, 0.3 mm inter-slice gap, 100 µm in-plane isotropic resolution (matrix size 300 px x 110 px, FOV 30 mm × 11 mm), three averages, and 210 kHz receive bandwidth. To measure the water-only signal, the second high-resolution scan was performed with the fat suppression module switched on (PDw_FS scan). In order to avoid bias errors, automatic receiver gain adjustment was bypassed, and the corresponding gain setting was manually copied from the first PDw scan. The scan duration was 6 min 45 s.

Fat fraction (FF) maps were calculated from PDw and PDw_FS images in FIJI/ImageJ software v1.53f (National Institutes of Health, Bethesda, USA) *via* a semi-automatic procedure using an in-house developed macro tool. The macro first reduces the user-defined interscapular ROI to tissue voxels only by thresholding. Next, the proton density fat fraction map was computed by dividing the fat-only image with the fat + water image, using the expression FF = SI_fat_/SI_fat+water_. This yielded a voxel-wise map of fat fraction ranging from 0 to 1. Notably, SI_fat_ in the above expression represented the calculated signal intensity of fat molecules only (SI_fat_ = SI_fat+water_-SI_water_ = PDw–PDw_FS). After the FF map was calculated, voxels were classified into BAT and WAT based on previously defined thresholds (BAT: 0.2 < FF < 0.7; WAT: FF > 0.7; thresholds were determined from *ex vivo* scans of excised BAT and WAT samples). Finally, automatic volumetric analysis with sample-wise averages and variances was computed using the combined variance formula, which takes into consideration different ROI sizes between slices and samples.

### Infrared thermography

The interscapular BAT activity was measured using a thermal camera (FLIR T-650sc, FLIR Systems, Wilsonville, OR, USA), as shown previously (Habek et al., 2020; Kordić et al., 2022). The experimenter was “blinded” to the group of animals that were scanned. The day before measurements, the animals were shaved between the scapulas. As previously described, to determine the activity of BAT in animals, we calculated BAT heat energy output before and after a meal, as well as after UGN, GLP-1, insulin, or linaclotide application, by using the Stefan–Boltzmann law.

In patients with diabetes, BAT activity was measured as previously described (Kordić et al., 2022). Before measurements, patients removed clothing from the upper part of the body for at least 30 min and rested for at least 1 hour. All measurements were performed during the summer months where the daily temperature was not less than 22°C to avoid cold-induced activation of BAT. The reflected temperature of the room was 25.9 ± 0.5°C, air temperature was 25.2 ± 0.4°C, and relative humidity was 56% ± 2%. BAT activity was presented as differences in maximal skin temperature above clavicles before and 1 h after a standardized meal (Graham bagel and yogurt). The activation of BAT was compared to basal skin temperature before a meal.

### Statistical analyses

To test the normality of the distribution, the Kolmogorov–Smirnov test was used. When UGN expression was compared between regions of the human PFC of subjects with an empty or full stomach, unpaired Student’s t-test was applied. For the analysis of UGN expression at the mRNA and protein levels in mouse brain samples, plasma and CSF glucose concentrations, and BAT activity, one-way ANOVA was used. To determine statistically significant correlation between glucose concentration in plasma and CSF with UGN expression in the mouse hypothalamus, cerebral cortex, and cerebellum, as well as activation of BAT after a meal and basal skin temperature above clavicles, we used Pearson’s correlation coefficient. The data were presented as mean ± standard error of the mean (SEM) or as median with interquartile range. P < 0.05 was considered statistically significant. For statistical analyses, GraphPad InStat statistical software (GraphPad Software, Boston, MA, USA) was used.

## Results

### Uroguanylin is expressed in the brain of both mice and humans

**FIGURE 1:**
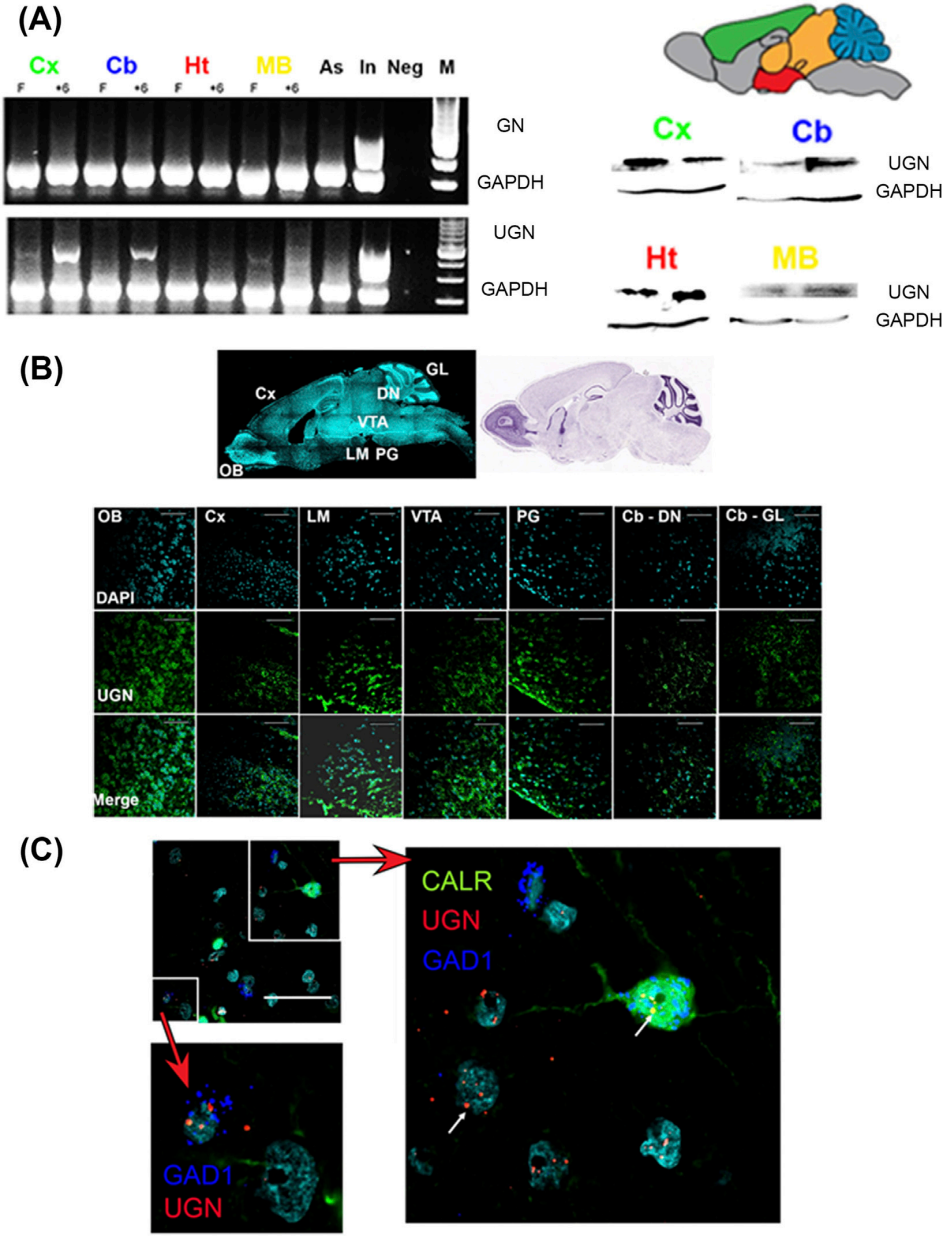
Uroguanylin was expressed in mouse and human brain. **(A)** Uroguanylin (UGN)-specific mRNA was found in different mouse brain regions. Samples from the cerebral cortex (Cx), cerebellum (Cb), hypothalamus (Ht), and midbrain (MB) were collected under fasting condition (F) and 6 h after a meal (+6). UGN was not expressed in primary astrocytes cultures (As). The positive control was UGN expression in the intestine (In) sampled 6 h after a meal. The negative control consisted of PCR without cDNA (Neg). The housekeeping gene used was GAPDH (glyceraldehyde-3-phosphate dehydrogenase). M, marker. ProUGN expression at the protein level was detected by Western blot. **(B)** Localization of UGN mRNA in the mouse brain was determined by RNAScope. DAPI staining of a sagittal section of the mouse brain and the corresponding Nissl staining (Allen Brain Atlas) are shown. In the olfactory bulb (OB), UGN was localized in the granular layer. UGN was also found in all layers of the somatomotor cortex (Cx), as well as in the granular layer of the cerebellar cortex (Cb-GL) and cerebellar deep nuclei (Cb-DN). In the midbrain and hypothalamus, UGN was localized in the ventral tegmental area (VTA) and the lateral mammillary nucleus (LM). UGN mRNA was also found in the pontine gray (PG). The bar represents 80 µm. DAPI - 4′,6-diamidino-2-phenylindole. **(C)** Uroguanylin mRNA was also expressed in the human prefrontal cortex. UGN mRNA was found in glutamate decarboxylase 1 (GAD1–RNAScope) and calretinin (CALR–labeled by specific antibody)-positive cells. The bar represents 50 µm. Arrows indicate UGN mRNA expression.

The mRNA for UGN (*Guca2B*) was detected in the mouse cerebral cortex, cerebellum, hypothalamus, and midbrain. UGN mRNA was not detected in pure primary astrocyte cultures. We confirmed that the PCR bands corresponded to UGN through sequencing and alignment analysis (Figure 1A). Expression of proUGN was confirmed at the protein level by Western blot analysis (Figure 1A) and immunohistochemistry. ProUGN was expressed in the mouse mammillary bodies, hypothalamus, and all cerebral cortical layers. The specificity of the staining was verified by the absence of specific signals in UGN KO mice (Supplementary Figure S1).

To localize UGN mRNA at the cellular level, we used *in situ* hybridization (RNAScope). In mice, UGN was expressed in the following: the granular layer of the olfactory bulb, all layers of the cerebral somatomotor cortex, the lateral mammillary nucleus, the ventral tegmental area, the pontine gray (pons), the granular layer of the cerebellar cortex, and deep cerebellar nuclei (Figure 1B).

We also confirmed UGN mRNA expression in the human brain. In the human PFC, UGN was diffusely expressed in neurons across all cortical layers of the superior frontal gyrus (BA9). UGN mRNA was identified in glutamate decarboxylase 1 (GAD1)-positive cells, a marker of GABAergic interneurons (Figure 1C), specifically in calretinin-, calbindin-, and parvalbumin-positive interneuron (Supplementary Figure S2). However, we cannot exclude the possibility of UGN expression in other types of cells within the human brain.

### Uroguanylin at the mRNA level in the mouse brain is regulated by feeding

**FIGURE 2:**
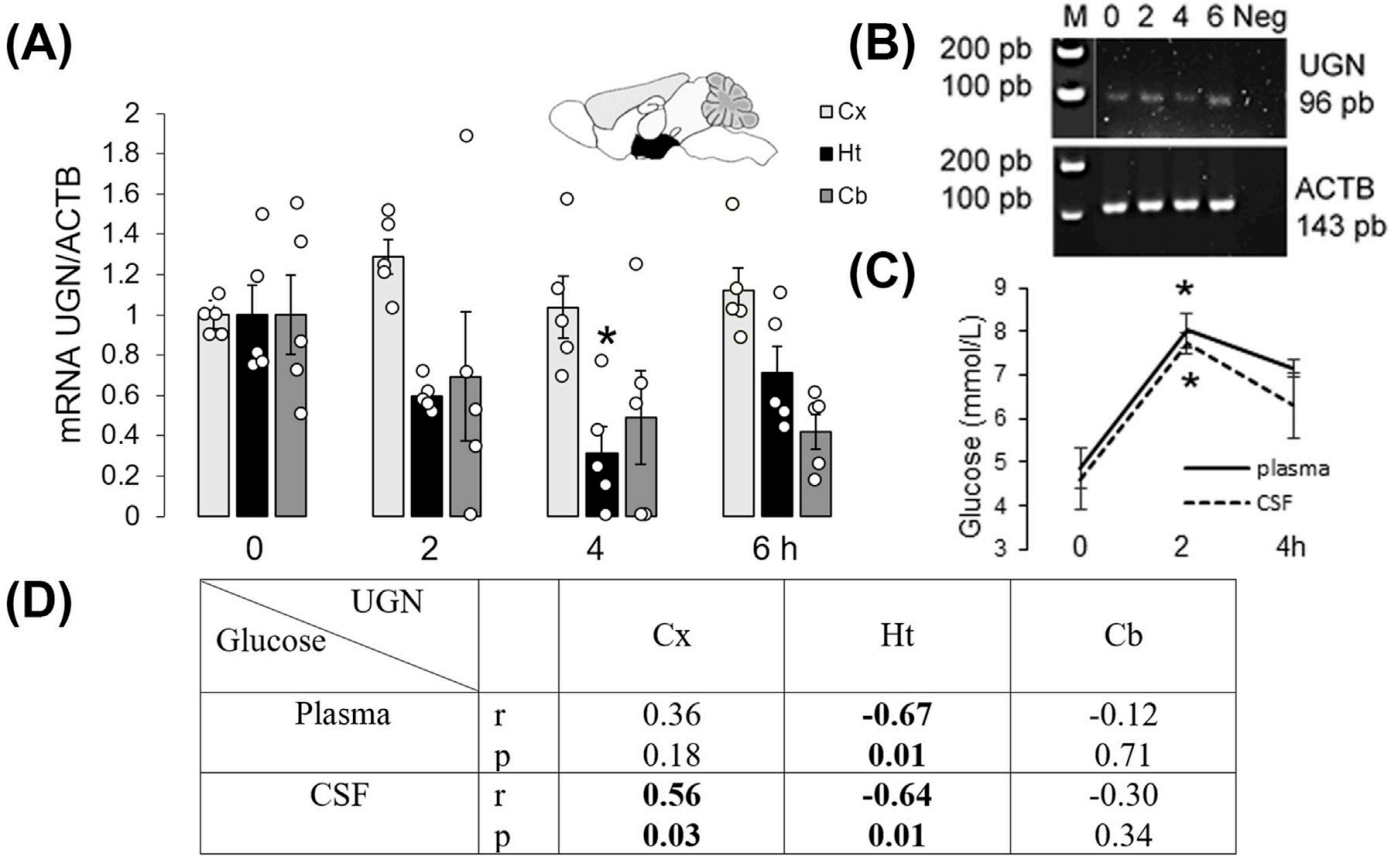
Uroguanylin expression in the mouse brain was regulated by feeding. **(A)** Changes in UGN mRNA expression levels were determined by qPCR, and products of this reaction are presented **(B)**. Control for the specificity of the qPCR included samples from the hypothalamus (Ht), where UGN – 96 bp and ACTB – 143 bp bands were detected. **(C)** Glucose concentration in plasma and cerebrospinal fluid (CSF) was measured. Results are presented as mean ± SEM. *p < 0.05 indicated a statistically significant difference (unpaired, two-tailed Student’s t-test, n = 5). **(D)** Two hours after a meal, UGN mRNA expression in the Ht was negatively correlated with plasma and CSF glucose concentrations, whereas in the Cx, UGN expression was positively correlated with CSF glucose concentration. Cb, cerebellum; Cx, cerebral cortex.

In the mouse hypothalamus, at the mRNA level, UGN expression decreased at 2 and 4 h after a meal (Supplementary Figure S3, original RNAScope staining). The summarized results are shown in Figure 2A. Two hours after a meal, mRNA expression increased by almost 30% in the cortex (p = 0.03, n = 5), whereas in the hypothalamus, it decreased by more than 40% (p = 0.03, n = 5) and remained unchanged in the cerebellum (p = 0.43, n = 5). The specificity of the qPCR products was confirmed by gel-electrophoresis (Figure 2B), and feeding was verified by measuring glucose concentration in the plasma and CSF (Figure 2C). Changes in UGN mRNA expression in the cerebral cortex 2 h after a meal were positively and significantly correlated with glucose concentration in the CSF, whereas in the hypothalamus, there was a statistically significant negative correlation between UGN mRNA expression and glucose concentrations in both the CSF and plasma (Figure 2D).

### Prouroguanylin expression is regulated by feeding, which is diminished in obesity

**FIGURE 3:**
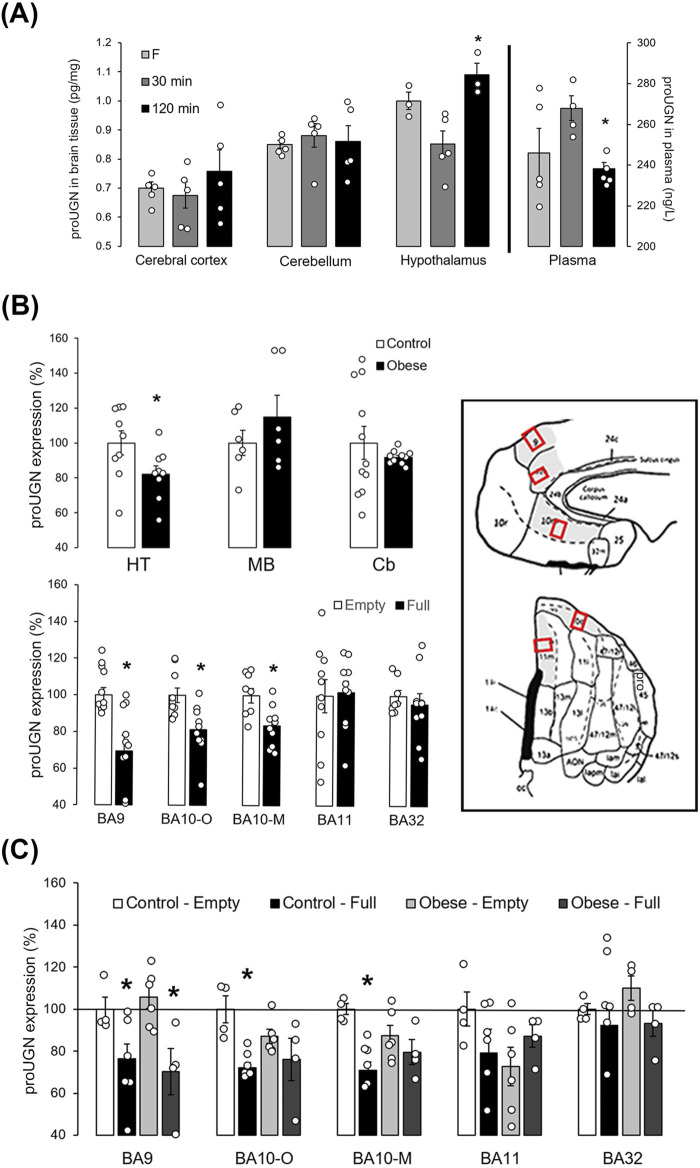
proUGN at protein levels was expressed in mouse and human brain and regulated by feeding. **(A)** After a meal, prouroguanylin (proUGN) expression was decreased in the hypothalamus (Ht), whereas at the same time point, proUGN concentration in plasma was increased (n = 3–5). **(B)** In the human Ht, expression of proUGN was decreased in individuals with obesity compared to those with normal body weight. There was no difference observed in the substantia nigra (midbrain–MD) or in the cerebellar cortex (Cb, n = 6–11). In addition, proUGN expression decreased in human Brodmann areas (BA) 9 and 10 (-O: orbital, -M: medial), but not in BA11 and BA32, in individuals with more than 200 mL (“full”) of stomach content (n = 8–10). **(C)** In individuals with obesity, the difference in proUGN expression due to fullness of the stomach was not present in BA10 (n = 4–6). Results are presented as mean ± SEM. *p < 0.05 indicated a statistically significant difference [**(A, C)** one-way ANOVA with Tukey’s *post hoc* test; **(B)** unpaired, two-tailed Student’s test].

To determine changes in the protein levels of proUGN after a meal in different brain regions and plasma, we used ELISA. Thirty minutes compared to 2 h after a meal, proUGN expression was decreased in the hypothalamus, whereas its concentration in plasma was increased (Figure 3A). Therefore, there was no positive correlation between plasma proUGN concentration and proUGN levels in the hypothalamus at any time point.

In the human hypothalamus, proUGN expression was lower in individuals with obesity than in the controls (normal body weight, Figure 3B). In the control group, proUGN expression decreased with age (r = −0.658, p = 0.039), whereas in individuals with obesity, this correlation was lost (r = −0.219, p = 0.571). When we examined the specific region of the human PFC involved in regulation of feeding, proUGN expression was decreased in BA9 and BA10 (orbital and medial parts) in individuals with more than 200 mL stomach content, classified as “full,” compared to those with less stomach content, classified as “empty,” as determined during autopsy. There was no difference in proUGN expression in BA11 and 32 (Figure 3B). The regulation of proUGN expression (empty stomach *versus* full) persisted in BA9 in individuals with obesity, whereas it was lost in BA10 (Figure 3C). These results suggest a highly specific regulation of proUGN expression in different PFC areas in humans, with varying responses to obesity.

### Acute and chronic activation of brown adipose tissue activity by centrally applied uroguanylin

**FIGURE 4:**
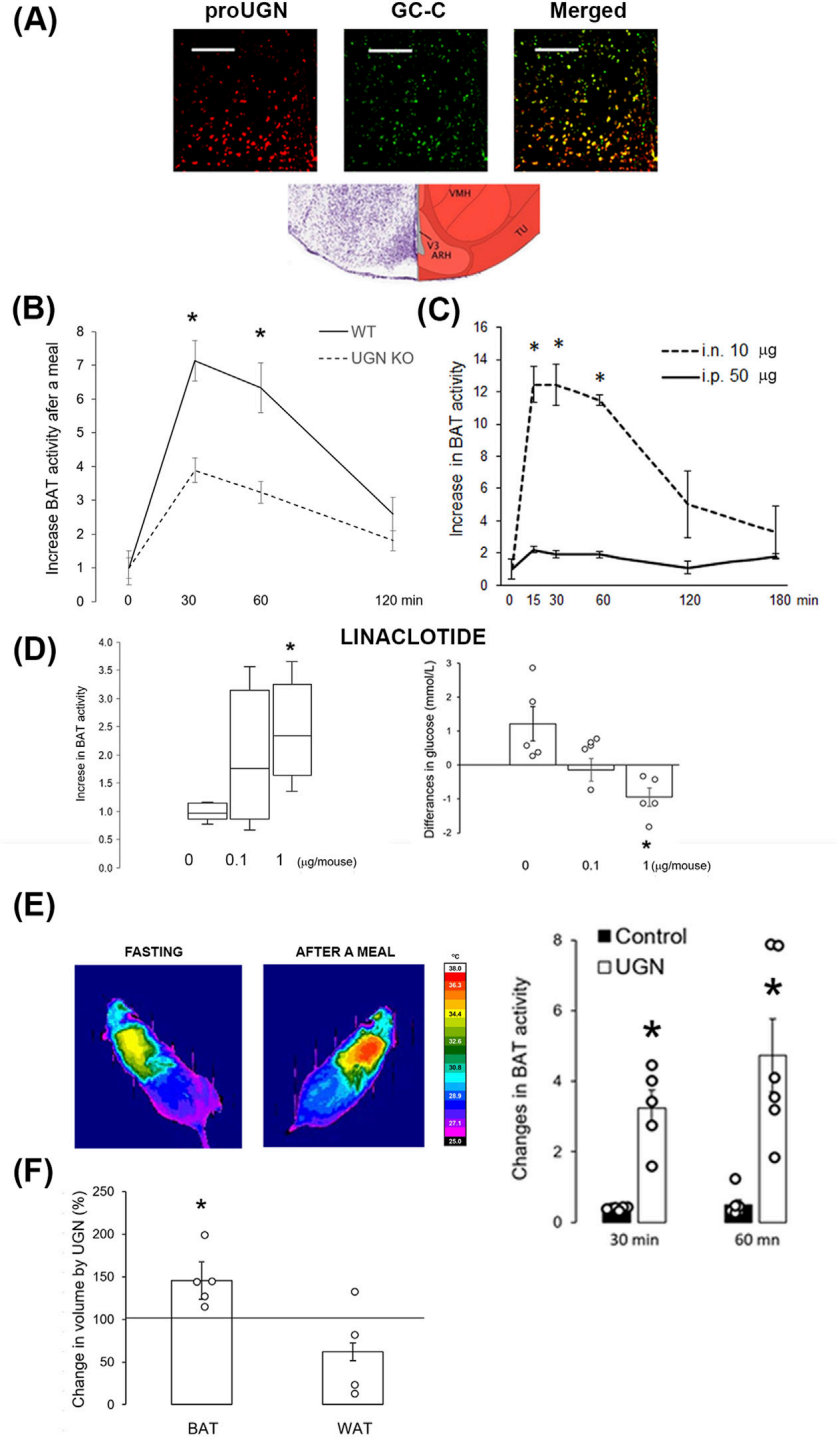
Intranasally applied uroguanylin and linaclotide increased BAT volume and activity. **(A)** Prouroguanylin (proUGN, red staining) was found near its receptor, guanylate cyclase C (GC-C, green staining), in the hypothalamic arcuate nucleus. The bar represents 100 µm. The Nissl staining and corresponding schema were modified from Allen Brain Atlas. ARH, arcuate nucleus; TU, tuberal nucleus; VMH, ventromedial nucleus of the hypothalamus; V3, third ventricle. **(B)** Increase in brown adipose tissue (BAT) activity after a meal was lower in UGN KO mice than in their WT littermates (n = 12). **(C)** Intranasal (i.n., 10 µg/animal) administration of UGN induced a six-fold increase in BAT activity at 15, 30, and 60 min after application compared to intraperitoneal (i.p., 50 µg/animal) administration (n = 4). **(D)** An increase in BAT activity was also observed with i.n. application of the UGN analog, linaclotide, 1 hour after application (n = 5). Results are presented as median with interquartile range. Linaclotide dose-dependently decreased plasma glucose concentration, shown as the difference in plasma concentration before and after linaclotide application, which corresponded to an increase in BAT activity. **(E)** Intranasal UGN application for 2 weeks increased BAT activation after a meal compared to its activation before treatment. Control, saline (vehicle). **(F)** After 2 weeks of i.n. UGN application, BAT volume increased in the interscapular region of the mice, which was measured by MRI. Results are presented as mean ± SEM. *p < 0.05 indicated a statistically significant difference; B, C, E, and F, unpaired, two-tailed Student’s test; D, one-way ANOVA with Tukey’s *post hoc* test.

Given the well-documented physiological role of GC-C in the arcuate nucleus of the hypothalamus in the regulation of feeding and BAT activity, we demonstrated the presence of proUGN (a precursor of UGN) near UGN’s receptor, GC-C (Figure 4A). To confirm previously reported effects of UGN on acute BAT activation after a meal, we conducted experiments on male WT and UGN KO mice (littermates). At 30 min and 1 h after a meal, WT mice exhibited a greater increase in BAT activity than UGN KO mice (Figure 4B). To investigate whether plasmatic UGN can affect hypothalamic UGN receptor, we applied a dose of UGN i.n. that was five times lower than the i.p. dose. I.n. application resulted in a more than 6-fold increase in BAT activity compared to i.p., lasting up to 1 hour after application (Figure 4C). The activation of GC-C in the brain was confirmed by a dose-dependent increase in BAT activity 1 hour after i.n. linaclotide (a GC-C agonist) application, which, at a dose of 1 µg/mouse, significantly decreased the plasma glucose concentration (Figure 4D).

After 2 weeks of i.n. UGN application (10 µg/animal/day), 5-month-old animals showed a greater increase in BAT activity after a meal (3.2 ± 0.5 and 4.7 ± 1.0 fold at 30 and 60 min after a meal, respectively) than in the activity before treatment (Figure 4E), at least partially due to an increase in BAT volume, as detected by MRI (Figure 4F). Interestingly, in animals treated only with the vehicle (saline), BAT activity declined (0.38 ± 0.02 and 0.49 ± 0.15 at 30 and 60 min after a meal, respectively), which is probably due to aging (Figure 4E).

### Regulation of UGN expression in hypothalamus

**FIGURE 5:**
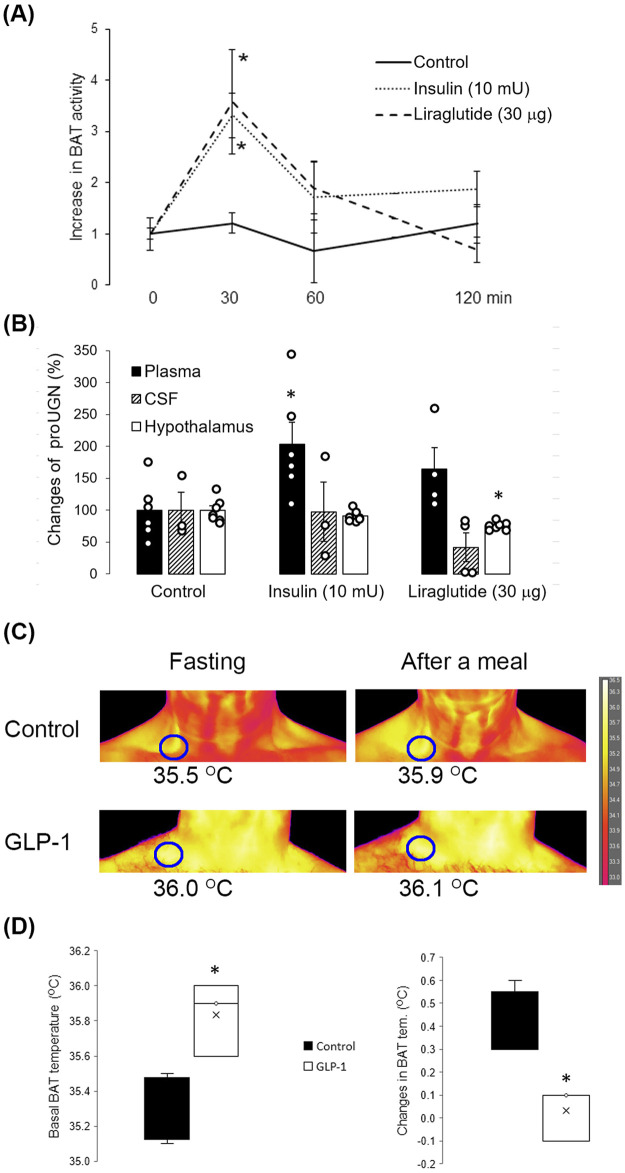
Effects of insulin and liraglutide on proUGN expression and BAT activity. **(A)** Thirty minutes after insulin and liraglutide intranasal (i.n.) application, BAT activity increased (n = 7). **(B)** Insulin increased proUGN plasma concentration but did not affect its levels in the cerebrospinal fluid (CSF) or hypothalamus. Liraglutide decreased proUGN expression in the hypothalamus but did not significantly alter its concentrations in the CSF and plasma. Results are presented as mean ± SEM (n = 3–7). **(C)** BAT activity after a meal in patients with type 2 diabetes (T2D), with or without GLP-1 therapy, is demonstrated in the original scans. **(D)** Basal temperature was higher, and the postprandial increase in BAT activity was lower in patients with T2D undergoing GLP-1 therapy than in patients with T2D receiving other types of therapy (control group, results are presented as median and interquartile range). *p < 0.05 indicated a statistically significant difference; **(A, B)** one-way ANOVA with Tukey’s *post hoc* test; **(D)** unpaired, two-tailed Student’s test (n = 4).

After a meal, GLP-1 and insulin are known to increase BAT activity. As both hormones are used in the therapy of T2D, we tested how they would affect the hypothalamic expression of UGN by i.n. (central) application of insulin and liraglutide (GLP-1 analog) in 2-month-old WT mice. After application, BAT activity increased as expected (Figure 5A). Two hours after insulin application, there was no increase in proUGN in the CSF and hypothalamus, but unexpectedly, there was a significant increase in proUGN concentration in plasma (Figure 5B). However, 2 h after i.n. application of liraglutide, proUGN levels decreased in the CSF and hypothalamus, which was not accompanied with the same change in plasma (Figure 5B).

As the analog GLP-1, liraglutide, decreased proUGN in the hypothalamus of mice, we tested whether GLP-1 therapy in patients with T2D could affect BAT activation after a meal. As shown in the original scans in Figure 5C, in patients with T2D who were not receiving GLP-1 therapy (control group), the maximal skin temperature above BAT increased by 0.4°C 1 hour after a meal. However, in a patient undergoing GLP-1 therapy, the fasting maximal temperature above BAT was higher than that in the control group, and there was no significant increase in BAT activity after a meal. The summarized results are shown in Figure 5D.

## Discussion

Patients with prediabetes and T2D have a reduced amount of supraclavicular BAT (Koksharova et al., 2017). After a meal, glucose uptake in BAT increases through both insulin-dependent and insulin-independent mechanisms (Orava et al., 2011). Therefore, insulin-dependent glucose uptake in BAT is diminished in patients with T2D (Hibi et al., 2016; Vosselman et al., 2013). However, the question remains as to which mechanisms are involved in postprandial BAT regulation and whether those mechanisms could be utilized to enhance BAT activity in patients with T2D as a new therapeutic approach for postprandial glucose regulation (Cheng et al., 2021). In this study, we demonstrate that postprandial activation of BAT was regulated by brain UGN. In mice, the GC-C agonist, linaclotide, enhanced BAT activity, which resulted in a decrease in plasma glucose concentration. Chronic application of UGN increased the BAT volume and postprandial BAT activation.

State of the art in measuring BAT activity is positron emission tomography–computed tomography (PET–CT) *via* accumulation of radiotracer fludeoxyglucose (^18^F-FDG). However, PET-CT fails as a method of choice for detecting the activation of BAT after a meal. One of the main reasons is that ^18^F-FDG competes for the same glucose transporter with endogenous glucose, which is increased after a meal, resulting in false-negative results which diminish the importance of BAT in health and disease. This should be taken into consideration, especially when we compared BAT activity in healthy individuals and participants with T2D with possible difference in blood glucose concentrations. The results should be standardized by blood glucose concentration, which is usually not measured before PET-CT scanning. Therefore, we used infrared thermography, which can detect the increase in BAT activity when the source of energy is glucose or fatty acids. The measurement itself is not affected by differences in blood glucose concentration (Kordić et al., 2022).

The existence of natriuretic peptides and their receptors in different parts of the mouse and human brain has been known for decades (Hodes and Lichtstein, 2014). The activation of membrane-bound and soluble guanylate cyclase leads to an increase in intracellular cGMP concentrations, which is involved in the regulation of synaptic activity in the hippocampus, amygdala, and cerebellum; modulation of noradrenergic neurotransmission; and neuroprotection against excitotoxic, metabolic, and oxidative damages as well as N-methyl D-aspartate (NMDA)-induced neurotoxicity (Kleppisch and Feil, 2009; Gong et al., 2011; Rodríguez Fermepín et al., 2022; Cao and Yang, 2008). The next step in the UGN signaling pathway involves cGMP-dependent protein kinase (PKG). Current evidence indicates that PKG activation promotes neural survival, proliferation, and migration of neural progenitor cells, synapse formation and plasticity (particularly in the hippocampus and cerebellum), nociception, and circadian rhythm (Fiscus and Johlfs, 2012). The role of cGMP and PKG in the hypothalamic regulation metabolism and glucose homeostasis remains unexplored.

UGN was expressed in brain regions that are involved in feeding regulation and BAT activation, as well as in the cerebellum and midbrain, which are involved in motor function. In the cerebellum, UGN was predominantly expressed in the granular layer, whose neurons form synapses with Purkinje cells, which are hyperpolarized upon GC-C activation by UGN (Habek et al., 2021). Similar expression has been observed for atrial natriuretic peptide (ANP) in the human cerebellar cortex (Kim et al., 2016). In the midbrain, dopaminergic neurons of the ventral tegmental area express GC-C, which could be activated by UGN synthesized in the same nuclei or by projections from other brain regions. The activation of this GC-C and an increase in intracellular cGMP could prevent the development of anxiety-like disorders through dopaminergic neurons projecting to the amygdala (Morel et al., 2022). The ventral tegmental area is also connected to the PFC, which expresses GC-C, including the dorsolateral prefrontal cortex (DLPFC), which is involved in the regulation of feeding behavior in humans (Hibi et al., 2016; Hosp et al., 2019; Settell et al., 2017). In the human PFC, UGN was expressed in different types of GABAergic interneurons (calretinin-, calbindin-, and parvalbumin-positive neurons), suggesting the existence of an additional subpopulation of UGN-positive interneurons. We are not surprised by this result, considering that many intestinal hormones are also expressed in interneurons, such as cholecystokinin and vasoactive intestinal peptide (Gonchar et al., 2008; Grieco et al., 2023).

The most investigated role of GC-C in the brain is its effect on glucose homeostasis and metabolism. As we identified UGN as the GC-C agonist in the brain, it is not surprising that in several mouse and human brain regions, UGN expression was regulated by feeding. Two hours after a meal, UGN mRNA expression was increased in the mouse cortex, with a positive correlation to glucose concentration in the CSF. At the same time point, there were no statistically significant changes in the protein levels of proUGN. However, when proUGN expression in the human PFC was compared between deceased subjects based on the fullness of the stomach, proUGN expression was lower in BA9 and BA10, but not in BA11 and BA32, when the stomach was full. BA9 is part of the DLPFC, and when this part of the PFC is active, it leads to overeating and obesity (Ester and Kullmann, 2022). BA10 is involved in the food evaluation process (Hollmann et al., 2013). After looking at pictures of food, the activity of BA10 decreased (Yoshikawa et al., 2016). We can speculate that UGN, along with the inhibitory effect of UGN-positive GABA interneurons, could be a part of this mechanism, as the expression of proUGN was reduced in this PFC region when the stomach was full. It is known that GABA is involved in the conscious choice of the amount and quality of ingested food (Farrar et al., 2008). As the feeding regulation of proUGN expression in GABAergic interneurons was diminished in obesity, the UGN-positive interneurons could be part of the mechanism by which GABA contributes to the development of obesity.

In the rodent and human hypothalamus, GC-C is localized in the arcuate nucleus on POMC neurons, whose activation by leptin and insulin leads to an increase in BAT activity. After a meal, POMC neurons enhance BAT activity *via* α-MSH (α-melanocyte-stimulating hormone) or through the sympathetic nervous system (Labbé et al., 2015; Tran et al., 2022). As i.n. administration of UGN was shown to increase the BAT activity and concurrently reduce the plasma glucose concentration (Habek et al., 2020), it is not surprising that the postprandial increase in the BAT activity is UGN-dependent, as evidenced by its attenuation in UGN KO animals. However, the exact mechanism of UGN signaling and the role of cGMP in POMC neurons remain to be elucidated.

The main agonist of hypothalamic GC-C is UGN synthesized in the brain but not derived from plasma. There are several reasons to conclude this: 1. i.n. application of UGN (absorbed into the CSF) at a dose five times lower than that of i.p. application increased BAT activity 6-fold; 2. chronic application into brain ventricles leads to an increase in BAT volume (Folgueira et al., 2016a); 3. proUGN was expressed near POMC neurons; 4. there was no positive correlation between proUGN in plasma and the hypothalamus or CSF after a meal; 5. radioactive proUGN, which is the predominant form of UGN in plasma and is cleaved to active hormone in target tissues, is filtrated by the kidneys but does not cross the blood–brain barrier (Qian et al., 2008).

Two hours after a meal, UGN mRNA expression in the mouse hypothalamus was decreased, and it was negatively correlated to glucose concentration in the CSF and plasma. At the same time point, changes at the protein level were opposite to the observed changes at the mRNA level. Differences in mRNA and protein expression at the same time point after an event are not new and are attributed to the different dynamics of mRNA synthesis and degradation, as well as protein synthesis (Moritz et al., 2019). Therefore, it was important to determine changes at both the mRNA and protein levels as sometimes protein expression does not immediately follow mRNA expression. In the human hypothalamus, there was a decrease in proUGN expression in individuals with obesity although obesity did not affect proUGN expression in the midbrain and cerebellum.

To investigate possible regulatory mechanisms of UGN expression in the brain, we selected insulin and GLP-1 (liraglutide) because both hormones can activate BAT shortly after a meal and are used in the treatment of patients with T2D (Lockie et al., 2012; Benedict et al., 2011). After i.n. application of insulin, there was no change in proUGN levels in the CSF and hypothalamus, whereas there was a significant increase in proUGN levels in plasma. Without adequate changes in the plasma concentration, i.n. application of liraglutide in mice led to a decrease in the hypothalamic proUGN expression, as was also observed in individuals with obesity. Furthermore, we tested whether BAT activity after a meal is affected in patients with T2D receiving GLP-1 therapy. The basal activity of BAT in the fasting condition was increased when compared to that in patients with T2D on other types of therapy. The possible reason for this is that GLP-1 therapy is not applied under physiological conditions with respect to feeding status as postprandial secretion of GLP-1 (30 min after a meal) is reduced in patients with T2D (Toft-Nielsen et al., 2001). Our results correspond to the previously published study by Janssen et al. (2020). When the basal activity was high in patients with T2D treated with GLP-1, postprandial BAT activation was decreased, which might result in low postprandial glucose consumption.

A limitation of this study is that experiments were conducted only in male subjects. Although sex and gender differences in UGN activity, BAT activation, satiety, glucose homeostasis, and obesity have been reported, these aspects were beyond the scope of the present study. However, future studies are planned to address these differences (Habek et al., 2020; Kroll et al., 2020). This is the first study to demonstrate the presence of UGN in both mouse and human brains. Furthermore, we showed that both acute and chronic administration of UGN in mice increases the BAT activity and volume and improves glucose homeostasis. Nevertheless, clinical trials to assess the efficacy, safety, and potential long-term effects of UGN in humans are currently not feasible. The clinical component of this study is limited by the small size number, but it suggests that GLP-1 agonists may influence the BAT activity, possibly through the modulation of UGN expression.

In conclusion, UGN is expressed in various brain regions of both mice and humans. In the hypothalamus, the expression of proUGN is regulated by feeding. This expression is reduced in individuals with obesity, which might reduce the potential effects of the active UGN hormone. In BA9 and BA10 of the human PFC, proUGN expression is decreased when the stomach is full, probably due to its degradation to the active UGN form. This effect still persists in BA9 of individuals with obesity, whereas it is not present in BA10. Although we still do not know the physiological importance of UGN actions in the PFC in individuals with obesity and T2D, centrally applied GC-C agonists increase the BAT activity, followed by a decrease in the plasma glucose concentration. Chronically, i.n. applied UGN also increases the BAT volume and postprandial BAT activation, which could be used as a new therapeutic strategy to increase BAT activity in patients with T2D for improved postprandial glucose regulation.

## Data Availability

The raw data supporting the conclusions of this article will be made available by the authors, without undue reservation.
