# Supplementary material for: Brain-derived uroguanylin as a regulator of postprandial brown adipose tissue activation: a potential therapeutic approach for metabolic disorders
Source: Front Pharmacol. 2025 Apr 25;16:1569163. doi: 10.3389/fphar.2025.1569163 (PMC12062040; doi:10.3389/fphar.2025.1569163)
Supplement: Supplementary file 1 [file DataSheet1.docx]

**Brain-derived uroguanylin as a regulator of postprandial brown adipose tissue activation: a potential therapeutic approach for metabolic disorders**

Nikola Habek, Martina Ratko, Dora Sedmak, Ivan Banovac, Vladiana Crljen, Milan Kordić, Marina Radmilović, Siniša Škokić, Martina Tkalčić, Anton Mažuranić, Pero Bubalo, Petar Škavić, Spomenka Ljubić, Dario Rahelić, Aleksandra Dugandžić

Table S1. List of deceased men included in this study.

| No. | Body weight | Age (years) | Cause of death |
| --- | --- | --- | --- |
| 1 | Participant with obesity | 57 | Heart failure |
| 2 | Participant with obesity | 59 | Heart failure |
| 3 | Participant with obesity | 34 | Electric shock |
| 4 | Participant with normal body weight | 39 | Acute poisoning |
| 5 | Participant with normal body weight | 67 | Thromboembolism of pulmonary arteries |
| 6 | Participant with normal body weight | 55 | Heart failure |
| 7 | Participant with obesity | 48 | Choking on a bite of food |
| 8 | Participant with obesity | 52 | Heart failure |
| 9 | Participant with obesity | 63 | Heart failure |
| 10 | Participant with normal body weight | 61 | Acute poisoning |
| 11 | Participant with normal body weight | 91 | Suicide |
| 12 | Participant with normal body weight | 41 | Suicide |
| 13 | Participant with obesity | 41 | Heart failure |
| 14 | Participant with normal body weight | 50 | Suicide |
| 15 | Participant with normal body weight | 31 | Heart failure and pneumonia |
| 16 | Participant with obesity | 74 | Choking on a bite of food |
| 17 | Participant with normal body weight | 40 | Acute poisoning |
| 18 | Participant with normal body weight | 67 | Internal bleeding |
| 19 | Participant with normal body weight | 66 | Heart failure |
| 20 | Participant with obesity | 60 | Cardiac tamponade |
| 21 | Participant with obesity | 31 | Suicide |


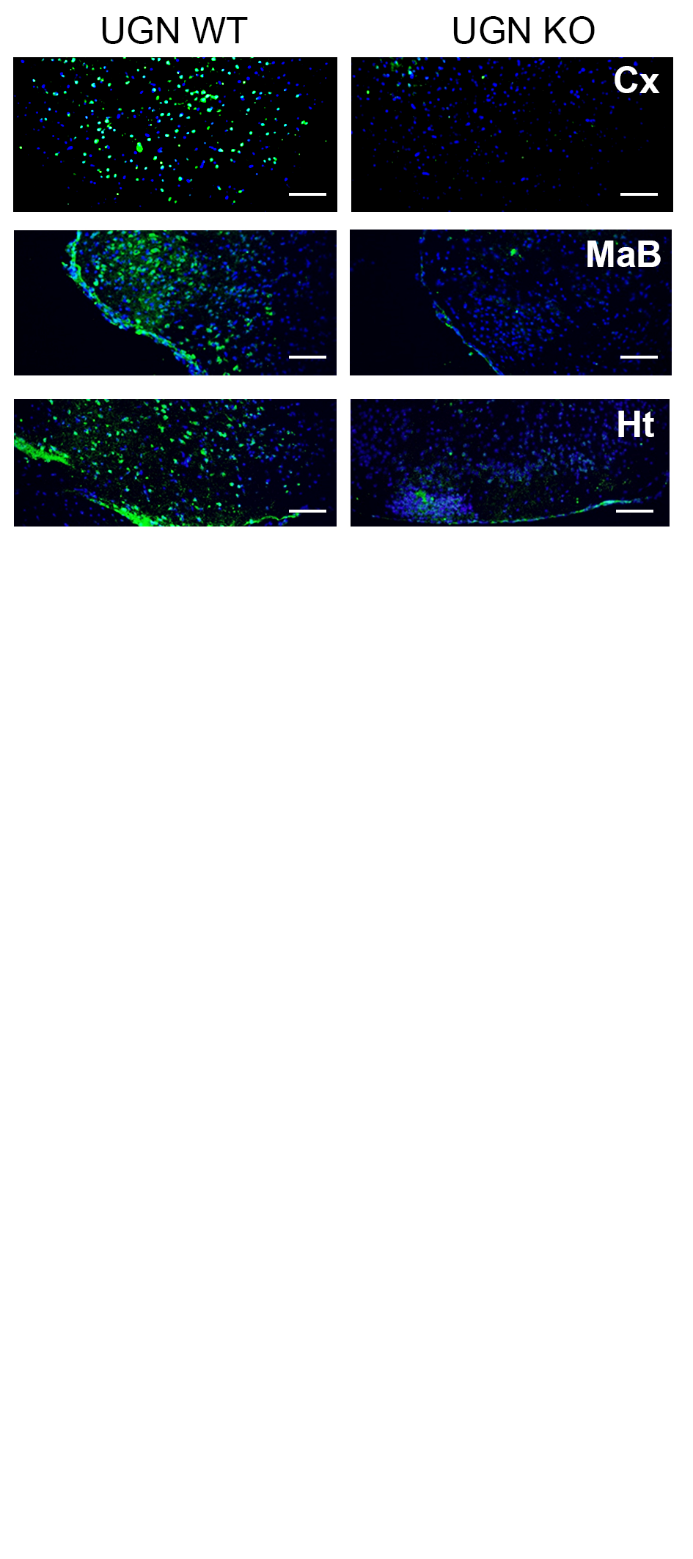


**Figure S1. Prouroguanylin expression at the protein level was found in cerebral cortex, mamillary bodies and hypothalamus.** The specificity of the immunostaining in WT mice was confirmed in UGN KO littermates where proUGN-specific staining (green) was not present. DAPI – blue staining. The bar represents 100 µm. Cx – cerebral cortex; MaB – mammillary body; Ht – hypothalamus

**
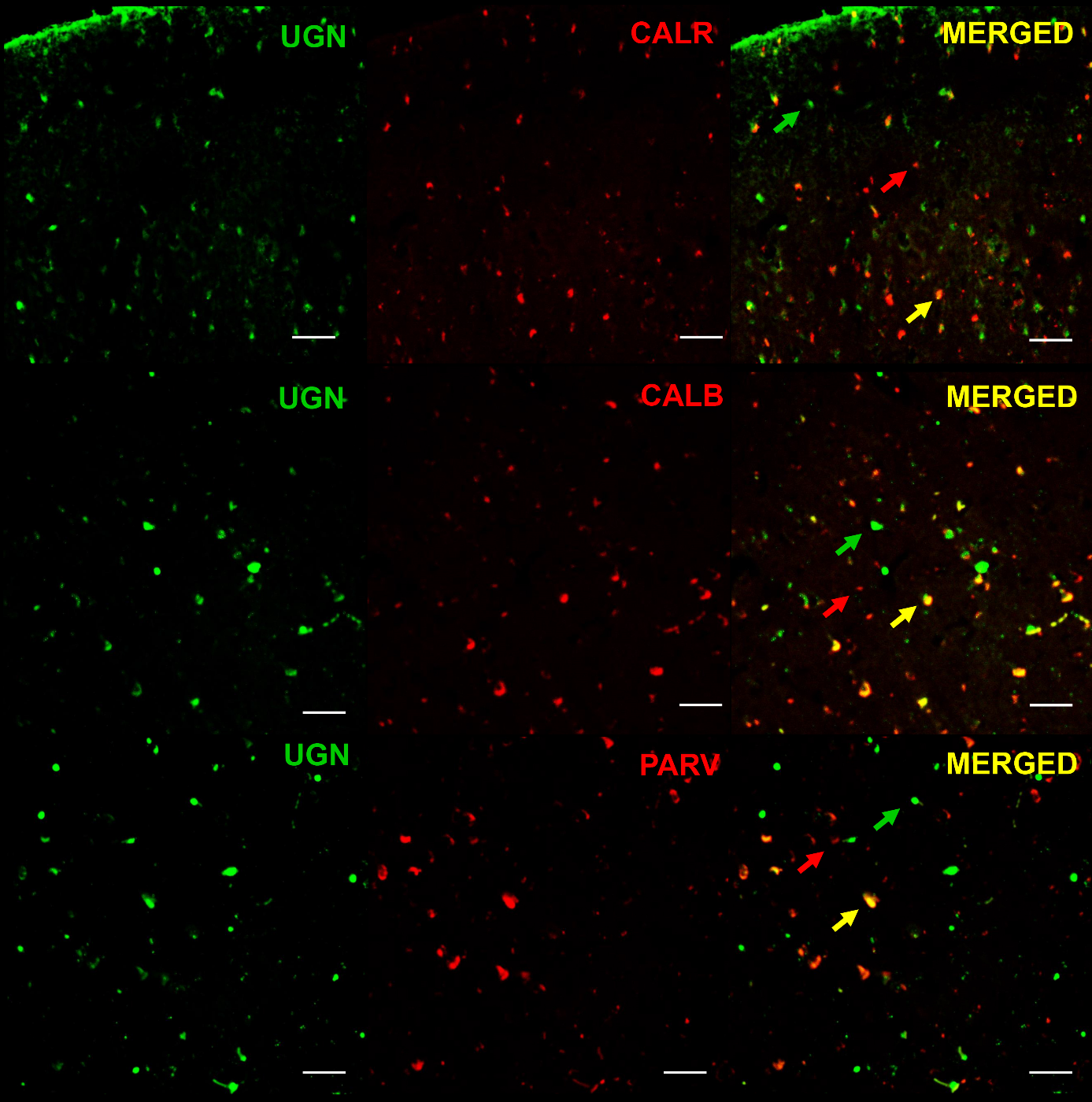
**

**Figure S2. Expression of UGN mRNA and markers of interneuronal subpopulations in the human prefrontal cortex.** Green arrows point to cells expressing only mRNA for UGN, red arrows point to cells expressing only markers of interneurons, and yellow arrows point to cells expressing both mRNA for UGN and markers of interneurons. The bar represents 50 μm. UGN – uroguanylin, CALR – calretinin, CALB – calbindin, PARV – parvalbumin.


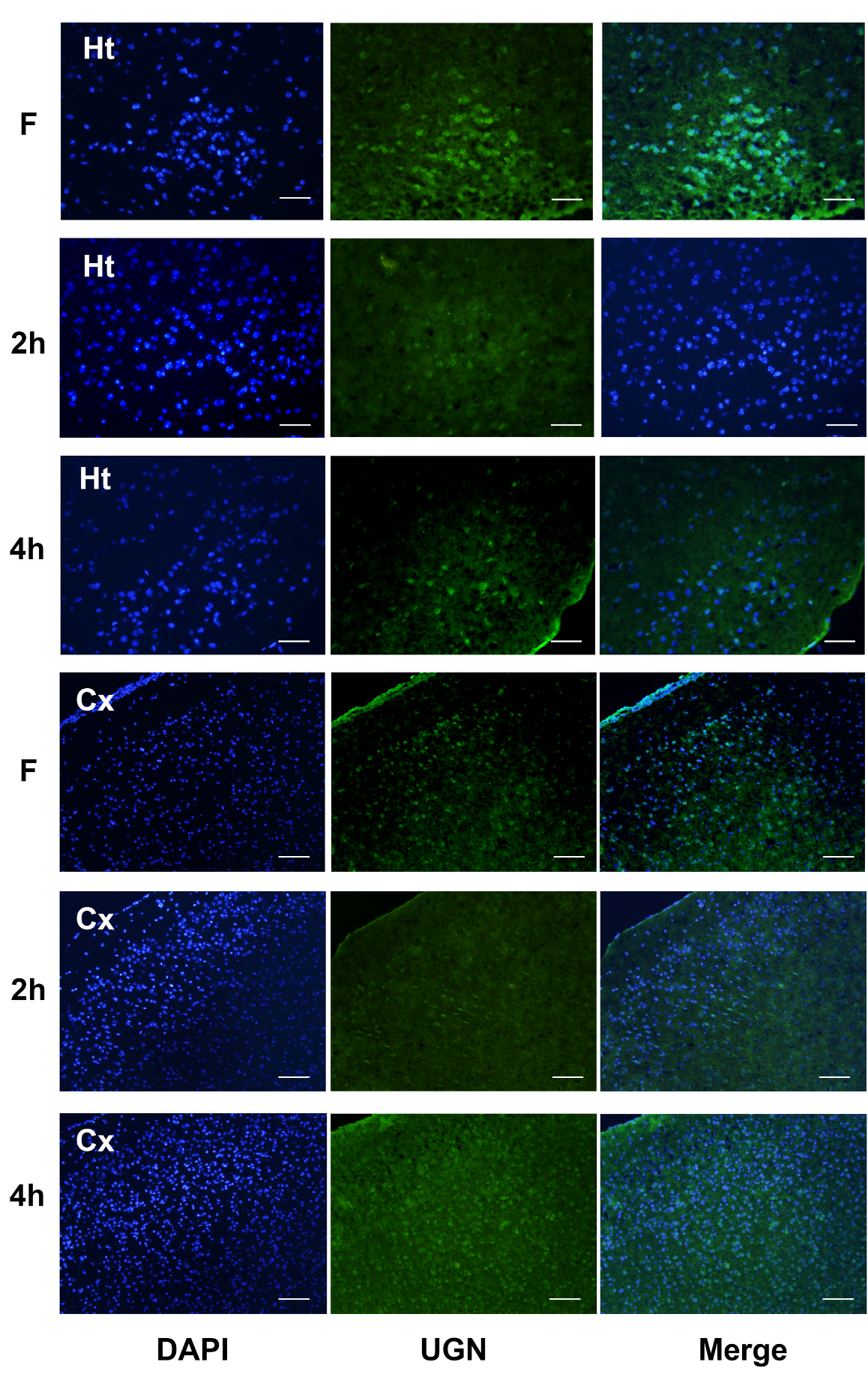


**Figure S3.** **Uroguanylin expression at the mRNA level in the mouse brain was regulated by feeding.** Uroguanylin (UGN) mRNA was found in the hypothalamus (Ht, bar = 40 µm) and the cerebral cortex (Cx, bar = 80 µm) of mice under fasting conditions (F) and 2 and 4 hours after a meal by *in situ* hybridisation (RNAScope).
